# Frequency-Based Representation of Massive Alerts and Combination of Indicators by Heterogeneous Intrusion Detection Systems for Anomaly Detection

**DOI:** 10.3390/s22124417

**Published:** 2022-06-10

**Authors:** Hyunjae Park, Young-June Choi

**Affiliations:** 1 Department of Computer Engineering, Ajou University, Suwon 16499, Korea; estancia@ajou.ac.kr; 2 Department of Artificial Intelligence, Ajou University, Suwon 16499, Korea

**Keywords:** intrusion detection systems, IDS alerts, information representation, situational awareness, machine learning

## Abstract

Although the application of a wide range of sensors has been generalized through the development of technology, the processing of massive alerts generated through data analysis and monitoring remains a challenge. This problem is also found in cyber security because the intrusion detection system (IDS) produces a tremendous number of alerts. Massive alerts not only significantly increase resources for analysis, but also make it difficult to analyze the overall situation of the system. In order to handle massive alerts, we propose using an indicator as a frequency-based representation. The proposed indicator is generated from categorical parameters of alerts that occur within a unit time utilizing frequency and is used for situational awareness with machine learning to detect whether there is a threat or not. The advantage of using indicators is that they can determine the situation for a period without analyzing individual alerts, which helps security experts to recognize the situation in the system and focus on targets that require in-depth analysis. In addition, the conversion from the categorical parameters which is highly related to analysis to numeric parameter allows for applying machine learning. For performance evaluation, we collect data from an HAI testbed similar to real critical infrastructure and conduct experiments using indicators and XGBoost, a classification machine learning algorithm against five famous vulnerability attacks. Consequently, we show that the proposed method can detect attacks with more than 90 percent accuracy, and the performance is enhanced using heterogeneous intrusion detection systems.

## 1. Introduction

The technical enhancement, cost reduction of sensors, and the development of the Internet of Things (IoT) have led to the wide adaptation of sensors. This huge trend has also triggered progress in the industrial domain to introduce the concept of a cyber-physical system (CPS) to achieve a higher level of operational efficiency and productivity as the objective of Industry 4.0 [1]. Several terms define the adoption of sensor networks in the industry, such as industrial control systems (ICS), Industrial Internet of Things (IIoT), CPS, and smart grid. Although each term describes a different aspect of this adoption in terms of scope, domain, or condition, it is clear that sensors can monitor industrial assets and production processes elaborately [2]. The IIoT monitoring architecture, which consists of a sensor monitoring agent, gateway monitoring agent, monitoring logger, management agents, and management system [3], is also generally adopted.

One of the widely known challenges in IoT is cyber security [4]. The common issues with IoT, such as privacy issues, vulnerability in protocols, authentication, and authorization, are still present in the industrial domain [5]. In addition, it is much more important in industry because the damage due to malicious attacks can be considerable compared to personal loss. However, the protocols in the industry are not designed to protect themselves from malicious attacks [6,7,8]. To defend this vulnerability in protocols, ICS applies an additional component, the intrusion detection system (IDS), which includes a function to detect systemic threats [9]. In order to detect threats, IDS obtains information such as system logs, host data, network traffics, and sensor measurements with domain knowledge. There are various types of detection rules in IDS. One is misuse-based detection, which checks whether there is any violation of the rules established by the domain knowledge. The other is anomaly-based detection, which identifies unusual behavior compared to normal status [8].

Many studies for intrusion detection have been published, and, recently, advanced machine learning and data mining techniques are also being employed in studies on IDS [10]. Although many studies improve the accuracy of detection by adopting machine learning models such as linear regression, clustering, k-nearest neighbor (KNN), and deep neural network (DNN), the problem of high false alert rate arises [11]. In addition, the tremendous increase in alerts causes difficulty in deciding the actual threat of the situation. Thousands of alerts in a short time require analysts to spend considerable time to conclude whether there is an occurrence of an actual intrusion or the alert is a result of excessive caution [12]. Some studies have attempted to extract alerts with pattern mining [13,14], correlation of alerts [15], flood sequence [16], etc. However, they also require supplemental setup as IDS reduces some false alerts even if it is set up appropriately. In industrial environments, IDS runs with a default setup in most cases [11]. In this situation, we explore a method to refine the massive alerts generated to other forms that can indicate the status of the period. Inspired by the histogram, which is used as an input of machine learning to illustrate the status of a network connection, we adopt statistical values as indicators.

In this paper, we reveal the problem of legacy IDS systems, especially the difficulty of situational awareness due to the large number of alerts generated from simulation environments using emulated attacks on the real IDS. In order to solve this problem, we propose a machine learning method to recognize a security threat based on statistical values of the IDS alerts within a specific time window. We verify the performance of the proposed method under simulation environments with real IDS products, compared with the result using alert information only. In addition, we propose a heterogeneous method that uses various alerts from heterogeneous IDSs. The contribution of this paper is summarized as follows:

- We propose a method to refine massive alerts in specific periods utilizing the frequency of categorical values as indicators to handle the non-numeric values in each alert.
- We introduce a method for situational awareness with the indicators applying machine learning techniques and evaluate the performance of indicators using the alert dataset obtained from a realistic testbed.
- We introduce the usage of a combination of indicators by heterogeneous IDSs and validate its result.

The remainder of this paper is organized as follows: In Section 2, we present related work and, in Section 3, we describe the proposed indicators for IDS, which is evaluated through experiments in Section 4. Finally, we present conclusions in Section 5.

## 2. Related Works

### 2.1. Alert Aggregation and Alert Correlation

One of the representative methods for dealing with massive alerts is alert aggregation. The similarity-based technique is the most widely used method in the domain of alert aggregation. The similarity between alerts is computed based on one or more fields such as the IP address of attacker and victim, ports, and types and timestamp of alerts. After the computation, alerts are reduced by clustering and aggregation based on the similarity. This reduction implies that similar alerts come from the same causes, and some of them can be neglected. A clustering technique is one of the most common techniques. The grouping of alerts that share the same root cause is introduced in [17], where the similarity of two alerts is defined to be the sum of the dissimilarity of their attributes. Lin et al. [11] proposed an improved multi-source heterogeneous alert aggregation method, which divided the alert aggregation features into attack mode constraints and time interval constraints, and different attack modes had different time interval constraints. The author used the relative mean-variance of the time interval proposed in [10]. The dynamic updating coefficient of time interval sets the average value of the time interval to the threshold of the initial time interval, thus making the time interval threshold adaptive to the change in the network attack environment. In [18], the combination of the K-mean algorithm with the genetic algorithm is proposed. It is applied to overcome the high dependence on the initial values that reach the local minimum of K-means. Although similarity-based approaches have less complexity in the algorithm, they also have some weaknesses. It is difficult to find a suitable metric of similarity, and the accuracy of similarity-based correlation analysis methods is not high enough. Another prominent approach is the pattern-based technique. This approach aims to find frequent patterns or sub-patterns to compress massive alerts. Pattern extraction is conducted based on association rule, sequential mining, or frequent episode mining. An improved version of the Prefix Span algorithm is applied by Brahmi and Yah [19] as a method of finding the most frequent patterns by distributing the alerts and their attributes in multi-dimensional tables. Sequential-based methods are useful for modeling and analyzing complex attack scenarios from sequences of individual events or steps that are a part of the same attack scenario. Pre/Post-conditions and graphs are also typical types of involved technologies [20]. The time-consuming nature of the model results from a large number of patterns being counted and matched by the system. Moreover, numerous redundant patterns are discovered with rare and interesting patterns.

### 2.2. Security Visualization

Security visualization is another research topic to deal with massive alerts. Security visualization is one of the specified domains which comes from data visualization to focus on security-related data. Even though security visualization does not particularly deal with alerts, some studies mainly consider IDS alerts. The shared goal of these studies is to support the analysis of alerts and situational awareness. Visual Firewall [21] visualizes firewall operations, IDS alerts, and overall network statistics through multiple views on a single screen. The statistics view illustrates the network’s overall throughput using a colored histogram. Ref. [22] has developed a novel visualization system, IDS RainStorm, to address the problem of the flourishing number of alerts generated from intrusion detection systems in large networks. The developed system consists of the main view that displays an overall representation of the network and a zoomed view that provides a detailed display of a user-selected range of IP addresses. Shiravi et al. [23] propose Avisa, a security visualization system that embraces a situation assessment component. The system assigns scores to hosts based on an accumulation of metrics that reflect the changes in various aspects related to the alerts received by a particular host in a monitored network.

As part of a method for dealing with massive alerts through the exploration of alert correlation and security visualization, we recognized the need for integration that can characterize multiple alerts. In addition, the frequency of alert occurrence and utilization of categorical information are necessary for a top-down approach that recognizes the overall situation in the context recognition process. Inspired by this, we propose a method for generating indicators that can represent the period as a way to recognize a situation by processing a massive alert, similar to alert aggregation with a time window. In addition, we introduce a practical use of indicators to achieve situational awareness using machine learning and extend the application of the proposed method to enhance the performance.

## 3. Method for Situational Awareness

In this section, we introduce the overall structure of the application for the situational awareness, the details of the indicators with machine learning for situational awareness, and extended practical use to enhance performance.

### 3.1. Overall Structure

As many studies have pointed out, the number of alerts coming from the IDS is usually substantial. It leads to difficulty recognizing the threat situation even though a security expert monitors the IDS alerts. The analysis requires support such as the information visualization [22,24] instead of solely monitoring the alerts to operate the analysis in time. Although studies related to data aggregation and studies on visualization have different aspects, they have similar security goals, the detection of intrusion or anomaly from massive alerts, i.e., the situational awareness. It inspired us to propose an indicator to represent the status of a specific period as a numeric value to be utilized by machine learning.

**Figure 1:**
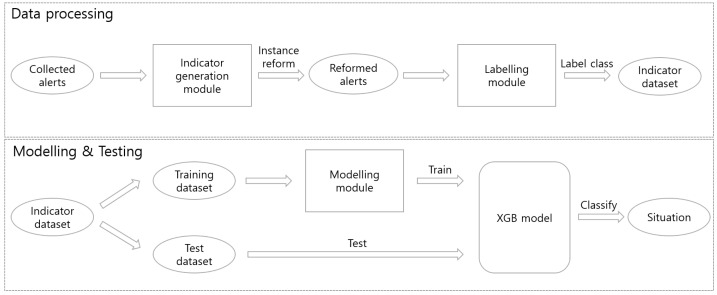
Process of situational awareness.

The framework of the proposed method to recognize security situations is found in classification with supervised learning. Therefore, it consists of data processing and modellng steps. Figure 1 shows the process of situational awareness. Our main idea for handling massive alerts is transforming input from alerts to indicators. This process incurs two modules: the indicator generation module and the labeling module. The indicator generation module generates a value according to the indicator generation rule using raw alerts as a value for each time period. The labeling module designates the class for the time–value pair created in the indicator generation module, and it is composed of normal or abnormal situations. We refer to this labeled result as an indicator dataset and use it as an input to create a classification model by applying machine learning techniques.

The modeling step is carried out using the indicator dataset created through data processing. The indicator dataset is a transformed in the form of the collected data and can be used as a training dataset, but in this study, it was divided into a training dataset and a test dataset for performance evaluation. For situational awareness with alert as an input value, it operates by generating an input value using the indicator generation module with the same settings as the data processing step. The modeling step uses training dataset to create a model like a typical machine learning technique. The generated model takes test data as input and returns a class for the situation. Consequently, we can represent situational awareness through the returned class.

### 3.2. Indicators

IDS keeps monitoring the network and generates alerts when when an anomaly occurs. Although the anomaly is generated with some margin, it should be treated as an actual anomaly. We were inspired by the fact that continuous monitoring of network packets and the generation of alerts as a result of monitoring can be handled in the form of time-series data. Although an alert is generated as a result of a monitored packet to be strictly classified, the accuracy of this result depends on the time during which the IDS analyzes and processes the packet. In other words, the relationship between the packet and the alerts can be replaced with that between the time and the alerts. For the implementation of situational awareness, which we aim for, the determination of a specific time or period is generally operated through analysis and classification of alerts for the time. Existing studies use alert aggregation, which is a method of excluding overlapping alerts or bundling multiple alerts with a specific pattern to deal with massive alerts in this analysis process, which is performed using detailed information on alerts.

**Figure 2:**
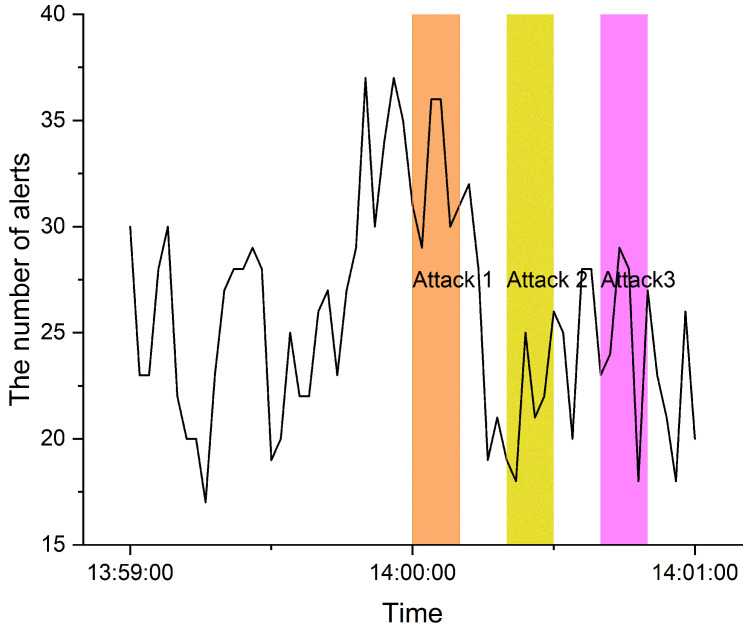
An example of the number of alerts.

We propose the concept of indicators in order to handle this problem. We assume that there is a link between the situation and the alert, even though IDS generates massive alerts, making the analysis difficult. Such correlation is generally performed through the analysis of individual alert information, but we approached it in terms of the frequency of alert occurrence, the commonality of multiple alerts, and the analysis of tendencies. An indicator is a concept introduced to analyze this point and is a numerical value that can represent the characteristics of multiple alerts. Indicators are statistical values based on the frequency of multiple alerts and represent characteristics in terms of the volume of multiple alerts at a specific time or period. The average and standard deviation of the alert frequency at a specific time is probably the most intuitive example. Since it was expected that the number of alerts could increase in the event of an anomaly, the initial trial focused on the frequency of all alerts. However, it is difficult to act on situational awareness simply through the frequency of alerts that occur. As shown in Figure 2, the frequency of alerts generated by IDS does not change significantly even in the existence of security threats.

In further exploration, we applied the indicator using the detailed field of the alert, commonly generated in every alert, such as the IP and port number of an attacker and victim. Since these fields are also generally used to analyze an alert, it was expected that it would be easy to understand the relationship between the situation and the alert. The challenge in analyzing these fields is that they consist of non-numeric values. Even though it is possible to use the non-numeric value itself with one-hot encoding or handling as categorical value, the treatment of category requires division as an individual case for each category. This still causes enough burden to demand the support of experts with each alert. Therefore, we explore the method to generate a value to be utilized with non-manual from non-numeric values of common fields. For *n* numeric values, the average value is generated by adding up the values of each numeric value and dividing by *n*. However, since it is impossible to operate between non-numeric values in data with *n* non-numeric values, it is burdensome to apply the average operation of numeric values values as is.

**Figure 3:**
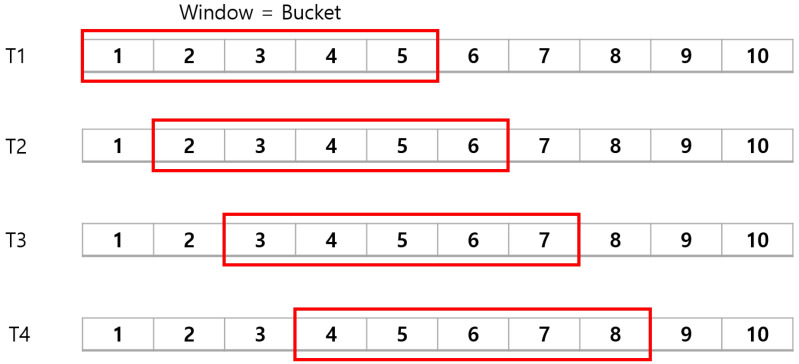
Example of sliding window as a bucket.

We propose the application of a value based on the frequency of occurrence of non-numeric values per unit time by utilizing the fact that alerts can be converted into values for time again. We attempt to aggregate data in the bucket of time-period to generate an indicator to represent the period. Specifically, an indicator representing the quantitative characteristics of non-numeric information of alerts in the bucket is generated by using the sliding window as a bucket for a time unit of a certain size. For example, as shown in Figure 3, statistical values are generated through information on all alerts for the time interval 1 to 5 for period T1 and used as an indicator for T1. In addition, the indicators for T2, T3, and T4 are sequentially generated as a value in which the sliding size, which is a predetermined unit time, increases.

The generation of an indicator using a non-numeric value is calculated using the frequency of the category of the non-numeric value of the target field within the bucket to be calculated. In the use of frequency, we impose a few changes in the statistical values to describe the tendency of the frequency of the alert. We apply five statistical values in order to generate the indicator: mean, standard deviation, skewness, kurtosis, and entropy. As shown in (1), we calculate the mean based on the frequency of each category within the target period. Here, we define $c_{i}$ as the frequency of each category *i*, and *k* is the number of categories in the period.

The generation of an indicator using a non-numeric value is calculated using the frequency of the category of the non-numeric value of the target field within the bucket to be calculated. In the use of frequency, we impose a few changes in statistical values to describe the tendency of the frequency of the alert. We apply five statistical values to generate the indicator: mean, standard deviation, skewness, kurtosis, and entropy. As shown in (1), we calculate the mean based on the frequency of each category in the target period. Here, we define $c_{i}$ as the frequency of each category *i*, and *k* is the number of categories in the period:

(1) $$Mean\left(X\right)=\frac{\sum_{i=1}^{k}F\left(c_{i}\right)}{k}=\bar{x}.$$

Similarly, we compute other statistical values following (2)–(4). In the case of the entropy, it requires the probability of each category. We calculate $p(x_{i})$ as the number of alerts in category *i* over the total number of occurred alerts:

(2) $$Std\left(X\right)=\sqrt{\frac{1}{n-1}\sum_{i=1}^{n}{\left(x_{i}-\bar{x}\right)}^{2}},$$

(3) $$Skewness\left(X\right)=\sqrt{\frac{\sum_{i=1}^{n}{\left(x_{i}-\bar{x}\right)}^{3}}{{\left(\sum_{i=1}^{n}{\left(x_{i}-\bar{x}\right)}^{2}\right)}^{\frac{3}{2}}}},$$

(4) $$Kurtosis\left(X\right)=\sqrt{\frac{\sum_{i=1}^{n}{\left(x_{i}-\bar{x}\right)}^{4}}{{\left(\sum_{i=1}^{n}{\left(x_{i}-\bar{x}\right)}^{2}\right)}^{2}}},$$

(5) $$Entropy\left(X\right)=-\sum_{i=1}^{n}x_{i}\log p\left(x_{i}\right).$$

**Figure 4:**
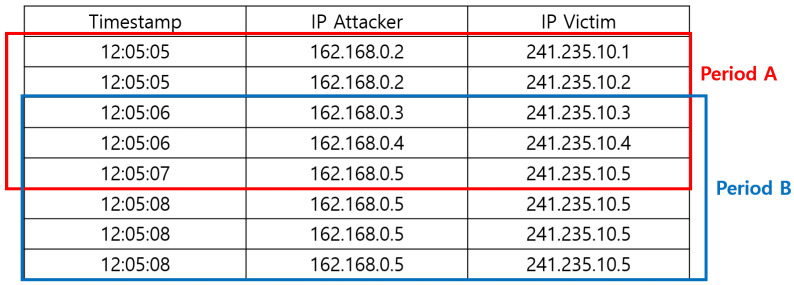
A simplified data sample to generate an indicator.

Figure 4 shows a simplified data sample for the generation of an indicator. The alert data have timestamp and category values. It has two non-numeric fields: IP Attacker and IP Victim. In cases when the window size is two seconds, the time period A is from time 12:05:05 to 12:05:07. Period A has five alerts, and there are four categories of IP attackers within the period. The mean of IP Attacker in period A is calculated as follows: The four categories, 162.168.0.2, 162.168.0.3, 162.168.0.4, and 162.168.0.5, have frequency values of 2, 1, 1, and 1, respectively. The frequency values for each category are summed and divided by the number of categories for the calculation of the types of categories existing in the bucket, which is equal to 1.25 by (2 + 1 + 1 + 1)/4 in the example. In entropy calculation, each category in IP Attackers has a probability of 1/5 except 162.168.0.2, which has 2/5. Therefore, the entropy in IP Attacker is 2*2/5 + 1*1/5 + 1*1/5 + 1*1/5 = 1.4. Similarly, their std, skewness, and kurtosis can be calculated. With this process, we derive a pair of the time and indicators for period A.

The proposed method to generate indicators is based on the time bucket, which can be generally considered as a time window. Hence, the sliding of the time window is the change of the bucket. In this example, period B is the next bucket when the slide size is one second. The generated indicator is a numeric value based on the frequency of alert categorical information included in the unit time section, and the value itself expresses the characteristics of the unit time section. We need another method, Section 3.3 for applying machine learning using the indicator, for situational awareness.

### 3.3. Label

Individual alert information is changed to a representative value for the period while the indicators are generated. The label of the situation is needed to adapt to the changes. In order to handle this, we need to decide how to match the attack and alert and how precise the threat situation is partitioned. It is impractical to precisely match the attack packet with an accurate identifier as an input, and the alert as a response to the packet. However, it is obvious that there is a relationship between the attack packet and the alerts, even though there is a time gap and incomplete reactions, such as incorrect alerts and failure to detect. Even if false alerts occur or a specific attack cannot be detected due to the performance of the IDS, the indicator represents information indicating the characteristics of the situation, so it is reasonable to determine the situation in the same IDS as learning.

Hence, we decide to consider every alert in the specific time period as a response to the attacks. In other words, we handle the alerts in time t as the response to the attack in time t even though there is a time gap between occurrences of the attack and the alert. We empirically checked that the time gap is not exceeding a few hundred milliseconds in our dataset except in a particular case. Although all IDSs monitor the same packet as input, the response from each IDS is unique in terms of alert type, frequency, and alert fields. The declaration of detailed attack type in alerts is not standardized and follows the IDS option. We classified the attack types into five categories in accordance with the attack scenario, which will be discussed in Section 4.2.

### 3.4. Model

A classification machine learning model is required for the situational awareness method using the proposed indicators. We choose to apply the XGBoost (XGB) method as the model, widely used in practice and is popularly agreed to provide high capability in classification performance compared with other machine learning models [25]. Among several deep learning methods for classification, we selected XGB because it shows the best performance and suitability for operator resources of tabular data by accommodating the indicator to situational awareness. There are several deep learning methods for classification but considering that the performance that generally surpasses XGB for tabular data does not come out [26], XGB, which has an advantage in terms of operator resources, was selected. The classification in XGB applies linear regression in the objective function and root mean square error in the evaluation metric. Although XGB supports multi-class classification, we decided to apply two-class classification considering that the accuracy of multi-class classification decreases compared to the two-class classification and considering that the focus of situational awareness is anomaly detection. Two-class classification classifies whether the situation is normal or abnormal (i.e., anomaly). We deploy a model for each attack class for situational awareness of various types of attacks. Table 1 shows the details of our setup.

### 3.5. Combination of Indicators from Heterogeneous IDS

We dealt with the creation and utilization of indicators for the unit time period by grouping alerts on packets. In this process, the form was changed to a time–value pair, and it was converted into situation information for a unit time bucket regardless of IDS. We propose a method to converge and utilize information among heterogeneous IDSs that can further improve performance. We found that the detection performance of each IDS varies and has pros and cons although the detection performance of each IDS is different. It is expected to enhance the effectiveness to utilize the indicators of heterogeneous IDSs together if each IDS monitors the same network. Because the alert of each IDS is information about the unit time period, the indicators from different IDSs can be utilized together. We have demonstrated experimentally improved performance of the combination of indicators from heterogeneous IDSs, which will be presented in the following section. Even though it is hard to install the real product, the additional utilization of virtual IDS is relatively easy. We expect that the mixture utilization of alerts in heterogeneous IDSs can be adopted in the form of a mixture of physical products and virtual tools in the field.

## 4. Experiments and Results

This section describes the experimental results and their interpretation.

### 4.1. Dataset

**Figure 5:**
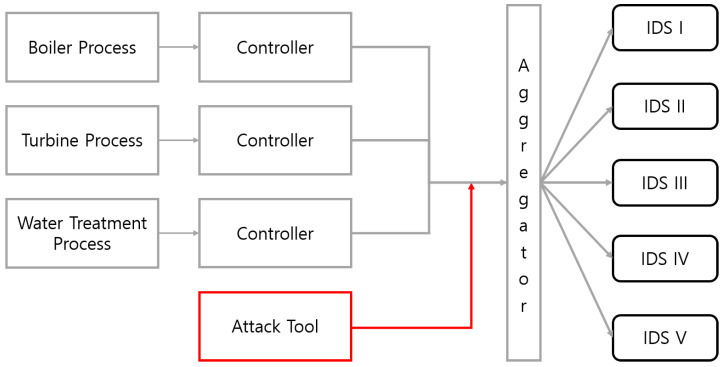
Outline of HAI testbed.

The dataset comes from the HAI testbed [27], which implements real control systems used for critical infrastructure. The testbed includes a physical attack tool, network attack tool, and network aggregator to collect events [28,29]. As shown in Figure 5, the testbed contains three systems: a boiler process, a turbine process, and a water treatment process. Each system collects data through a separate controller, and the data collected from each controller are passed to an aggregator. An attacker tool forges a physical attack by sniffing the transmitted data or creates a vulnerability attack. The aggregator delivers the aggregated data including attack data to each IDS to perform detection. As a result of these steps, the alerts are generated from the commercial IDSs and open source-based IDSs with an attack scenario. Table 2 shows the details of IDSs.

### 4.2. Configuration of Attack Scenario

We select attacks in order to generate the alert dataset following the attack scenario in the control systems. Attackers who aim to execute offensive actions to control systems must obtain information on system operation and environment. If they do not acquire prior information about the system, exploration of assets is generally the first step in obtaining the information about the system. After the asset exploration, searching the service, discovering the vulnerability, and attacking the weak point can be executed in a sequence. Otherwise, attackers can choose to execute the Denial of Service (DoS) attack in case they cannot find the vulnerability or every other attack fails. We designed a realistic scenario to attack the control system in the testbed and followed the above-mentioned steps against the attacks. The selected target of attack and the attack methods are listed as follows:

- Web-based Management. The Wireless HART gateway and system for web management are targeted by attacks to HTTP vulnerability;
- Database. Database server is targeted by attacks to database vulnerability;
- Operating System. Engineering Workstations (EWS) and Operator Workstations (EWS) based on window OS are targeted by attacks to SMB vulnerability;
- Remote Procedure. Modules for remote control and procedure call are targeted by vulnerability attacks such as DCERPC and VNC.

In the scenario, attackers first explore the assets and the system environments. Then, they try vulnerability attacks to target Web-based Management, Operating systems, Database, Remote Procedures, ICS-specific Protocols, and Time Synchronization in sequence. After every vulnerability attack, Distributed Denial of Service (DDoS) is performed. Table 3 provides information on the attacks included in the scenario. The attacks included a total of 316 vulnerabilities and malware attacks and 13 types of DDoS attacks. Details of each type of attack are as follows. There are 152 types of HTTP attacks, 18 types of MySQL attacks, 81 types of SMB attacks, and 65 types of DCEPRC attacks. The interval between each vulnerability attack is 10 s from the start of each attack. The DDoS attack has an attack time of 5 min for each type, and has an interval sufficient to resolve the delay problem of the logs.

### 4.3. Results

Before evaluating the classification performance, we introduce the reduction of the number of instances through data processing using indicators. As shown in Table 4, each attack generates a large number of alerts according to the IDS. Before applying alert aggregation, the number of each alert is the same as the number of instances during training, but when an indicator is used, it has a value for each time window, thus generating instances proportional to the amount of time. As a result, for each attack, HTTP, MySQL, SMB, DCEPRC, and DDoS have 757, 70, 404, 327, and 1950 instances, respectively. The normal class was collected for the period one hour before the attack to represent the section other than the attack. In evaluation of classification performance, we used accuracy as a performance matrix with 5-fold cross-validation. The entire dataset is divided into five bundles and one of the bundles is selected as a test set and the other ones become training sets in the 5-fold validation process.

**Figure 6:**
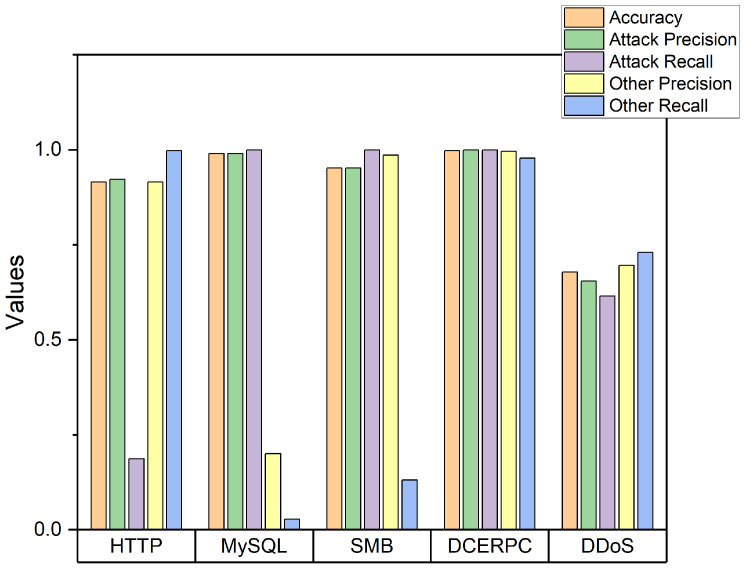
Performance of the unbalanced dataset.

In evaluation of classification performance, we used accuracy as a performance matrix with 5-fold cross-validation. The entire dataset is divided into five bundles and one of the bundles is selected as a test set and the other ones become training sets in the 5-fold validation process. In addition, we evaluated the performance with refined data randomly selected that is one-on-one matched to comparing classes to avoid a bias toward a specific class. For example, in testing a model for two-class classification for whether it is normal (i.e., non-attack) or abnormal (i.e., attacks), the data in the attack class are selected equally to normal from HTTP, MySQL, SMB, DCEPRC, and DDoS. If the model uses entire data from the dataset, which means that the class of normal data in the entire dataset occupies a large portion of train and test data, it brings biased results. These biased results improve accuracy, which is one of the performance evaluation indicators, whereas precision and recall are low. Such results are found in Figure 6, which shows the performance of IDS III when the data between the two classes are not balanced. In the case of HTTP, MySQL, SMB, and DCEPRC, the accuracy is all higher than 0.9, but in the case of HTTP, the recall of attacks is 0.187, which is very low, and in the case of MySQL, the precision and recall of others are 0.2 and 0.028, respectively. It indicates that increasing the accuracy of the attack class may give a bias in the classification because normal cases are treated as an attack even if they are not an attack.

**Figure 7:**
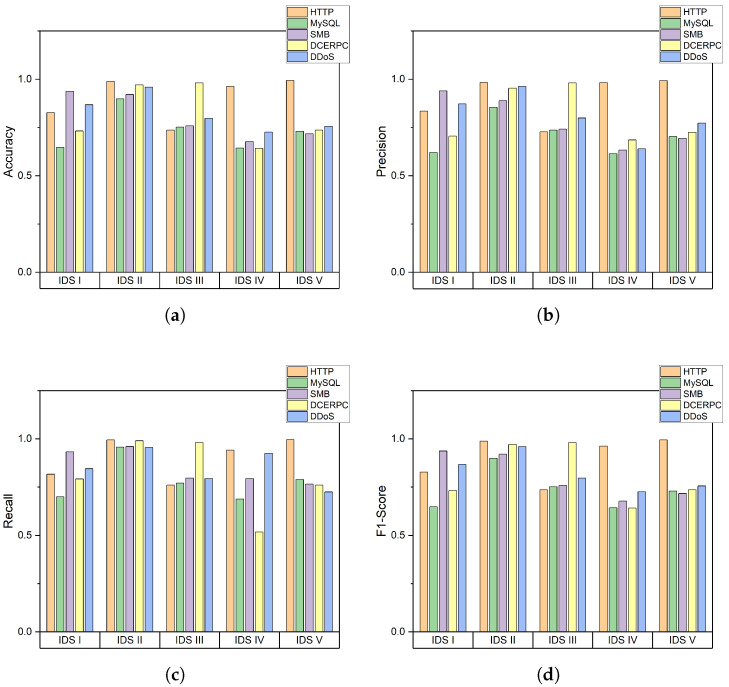
Performance of classification for each attack type: (**a**) Accuracy; (**b**) Precision; (**c**) Recall; and (**d**) F1-Score.

Therefore, in the following experiments, we applied a method of balancing the number of data between attack and non-attack classes during training. The accuracy of classification for each attack and each machine is exhibited in Figure 7a. The accuracy of classification for each IDS is above 90 percent, except for DDoS attacks. Even though there is some difference in overall performance among IDSs, IDS II exhibits the best accuracy, 0.988 in HTTP, 0.970 in DCEPRC, and 0.96 in DDoS attack type. Other IDSs also show that each of them detects certain attack types very well; e.g., IDS I detects SMB with high accuracy (0.936), IDS III detects DCEPRC (0.981), and IDS IV and IDS V detect HTTP (0.962 and 0.994, respectively). Because the indicators are refined parameters from alerts, their performance depends on the detection performance. These results provide an insight that integrating numerous alerts gives good performance in detecting attacks from heterogeneous IDSs.

The additional performance evaluation metrics such as precision, recall, and F1-score are shown in Figure 7b–d, respectively. The precision has a higher value as the ratio of the prediction by the algorithm to the actual correct answer is higher. Precision represents the ratio of the prediction by the algorithm to the actual correct answers, and recall represents the ratio of correct guesses made by the algorithm among the actual positive values. Both precision and recall evaluate the performance of how well the algorithm predicts positives, and both performances are expressed by F1-score at once. IDS II shows the highest F1-score and F1-score of each attack is 0.98, 0.89, 0.92, 0.97, and 0.95. In contrast, MySQL in IDS I and DCERPC in IDS IV show a low F1-score of 0.64. Through the above results, we confirm that two-class classification using indicators work well without any bias toward any class through precision, recall, and F1-score. Furthermore, each IDS detects a specific attack type with a high performance.

**Figure 8:**
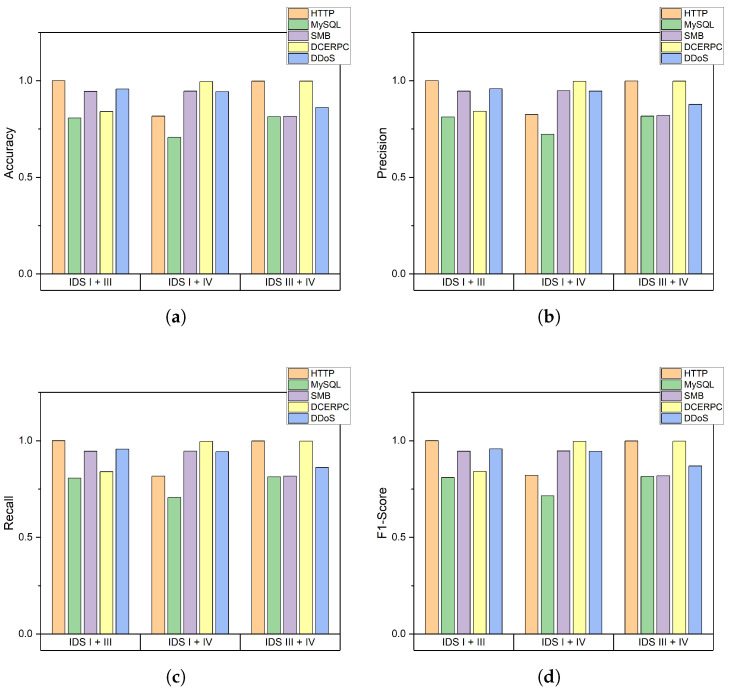
Performance of classification for each attack type in the setup of combination of heterogeneous IDS: (**a**) Accuracy, Accuracy of Heterogeneous IDS field; (**b**) Precision, Precision of Heterogeneous IDS field; (**c**) Recall, Recall of Heterogeneous IDS field; and (**d**) F1-Score, F1-Score of a Heterogeneous IDS field.

To explore further performance enhancement with the same dataset, we attempt to extend the proposed method for heterogeneous IDSs. IDS I shows high accuracy in SMB and DDoS attack but not in other attacks, while IDS III has high accuracy in DCERPC, and IDS IV and IDS V have high accuracy in HTTP attack types. Figure 8 shows the accuracy of classification with a combination of indicators of different IDSs, especially IDS I, IDS II, and IDS IV. By using both indicators of each IDS, it is expected that the performance is improved by the strong points of each IDS. The results in Figure 8 confirm further enhancement in performance. The accuracy of every class is enhanced over the highest performance of each IDS. The accuracy of HTTP attacks in the combination of IDS I + IV becomes 1, and in the combination of IDS III + IV becomes 0.998. Both combinations achieve an accuracy higher than 0.962 that of IDS IV. The performance of other classes is also improved. We conclude that the combination of indicators of different IDSs enhances performance.

## 5. Conclusions

IDS generates tremendous alerts, and representation of such alerts remains a challenge for anomaly detection. To handle this issue, in this study, we proposed a method to classify the state of the selected period by generating indicators with IDS alerts. The indicators are statistical values based on the frequency of alerts from IDS to represent a selected period. Through experiments that apply the practical alert data from the testbed, including real IDS products and attack tools to explore the feasibility in a realistic environment, we revealed that the refinement of alerts based on frequency could be utilized as a meaningful value. Furthermore, the experiments confirm that a combination of alerts in IDSs that monitor an identical packet improves the performance of classification. This method can be applied in fields wherein virtual IDS are installed in addition to deployed commercial IDS products. In future works, it is worth (i) determining the labels in the classification of threat situations considering the time gap and relationship of alert data, and (ii) exploring the deeper correlation between the indicators and alerts.

## Figures and Tables

**Table 1:** XGBoost setup.

Item	Setup
Model	Classifier
Max depth	4, 6, 8, 10
Learn rate	0.1, 0.15, 0.2, 0.01
Selected Fields	protocol, IPAttacker, IPVictim, portVictim, portAttacker
Window size	30
Sliding size	2

**Table 2:** Collectable security logs from five systems.

ID	Vender	Type	Installation	ACL	Signiture
I	A	IDS/IPS	Physical	✓	✓
II	B	IDS/IPS	Physical	✓	✓
III	C	IDS/IPS	Physical	✓	✓
IV	Open-source	Snort	Virtual	✓	✓
V	Open-source	Suricata	Virtual	✓	✓

**Table 3:** Summary of attacks in scenario.

Category	Service	Affected Testbed			Type	Attack Duration
		Boiler	Turbine	Water		
Web	HTTP	✓		✓	152	25 min 14 s
Database	MySQL	✓			18	2 min 21 s
OS	SMB	✓	✓	✓	81	13 min 29 s
Remote Procedure	DCERPC	✓	✓		65	10 min 55 s
DDoS					13	1 h 5 min
Total					329	1 h 56 min 59 s

**Table 4:** Instance reduction of applying indicators.

Class	Number of Alerts					Number of Instances with Indicators
	IDS I	IDS II	IDS III	IDS IV	IDS V	
HTTP	4056	25,062	281,515	31,845	3605	757
MySQL	336	2132	25,725	397	23	70
SMB	2262	11,778	148,418	2315	141	404
DCERPC	1610	10,043	122,927	3488	135	327
DDoS	17,174	99,268	632,560	197,090	140,348	1950
Normal	8819	51,082	658,427	11,903	493	1800

## Data Availability

Not applicable.
